# Paeoniflorin Attenuates Zinc Oxide Nanoparticle-Induced Pyroptosis in HepG2 Cells via the Activation of SIRT1/TFEB Signaling Pathway

**DOI:** 10.3390/antiox15101284

**Published:** 2026-10-03

**Authors:** Xingyao Pei, Zibin Li, Chun Chen, Wenjuan Zhang, Shang Wang, Yuefei Sun, Chongshan Dai, Daowen Li

**Affiliations:** 1 Tianjin Key Laboratory of Agricultural Animal Breeding and Healthy Husbandry, College of Animal Science and Veterinary Medicine, Tianjin Agricultural University, Tianjin 300392, China; peixingyao@tjau.edu.cn (X.P.);; 2 State Key Laboratory of Veterinary Public Health and Safety, Department of Pharmacology and Toxicology, College of Veterinary Medicine, China Agricultural University, Beijing 100193, China; 3 Department of Bioengineering, College of Life and Health, Dalian University, Dalian 116622, China; lizibin@dlu.edu.cn

**Keywords:** paeoniflorin, ZnO nanoparticles, pyroptosis, autophagy-lysosome, SIRT1, TFEB

## Abstract

Zinc oxide nanoparticles (ZnO NPs) have been applied in biomedicine, food processing, and agricultural production. ZnO NPs can cause potential hepatotoxicity in animals and humans; however, the precise molecular mechanisms remain unclear and the effective treatment strategies are scarce. In this study, we investigated the protective effects of paeoniflorin (PF), a bioactive compound extracted from the roots of Paeoniaceae plants, on ZnO NPs-induced hepatotoxicity using a cell model. Our results showed that PF supplementation can dose-dependently attenuate ZnO NPs-induced decreases in cell viability and oxidative damage in HepG2 cells. Mechanistically, PF significantly reduced the activation of NLRP3 inflammasome, then reduced the expression of GSDMD-NT cleavage and the secretion of IL-1β and IL-18, and finally ameliorated ZnO NPs-induced pyroptosis. Furthermore, PF can directly interact with SIRT1, then deacetylate TFEB and promote autophagy-lysosome biogenesis. Overexpression of TFEB markedly attenuated ZnO NPs-induced pyroptosis. Consistently, the inhibition of SIRT1 or knockdown of TFEB both partly attenuated the protective effect of PF. Taken together, for the first time, our study demonstrated that PF can directly target SIRT1, then effectively attenuate ZnO NPs-induced pyroptosis via the activation the SIRT1/TFEB signaling pathway.

## 1. Introduction

Zinc is an essential trace element whose intracellular homeostasis is tightly regulated by zinc transporters (ZIP/ZnT families) and metallothioneins (MTs), which are cysteine-rich proteins that buffer zinc through metal-responsive transcriptional induction [1,2]. However, when zinc uptake exceeds the MT-buffering capacity, the pool of labile zinc expands. This triggers reactive oxygen species (ROS) generation, mitochondrial dysfunction, glutathione depletion, and activation of inflammatory pathways, ultimately leading to cell death [3,4,5]. It has been demonstrated that liver is the primary organ for zinc accumulation and excessive accumulation can induce oxidative and inflammatory injury [6,7]. Zinc oxide nanoparticles (ZnO NPs) have attracted significant attention in various scientific and technological domains due to their unique physical and chemical properties, including a high surface area, photostability, biocompatibility, and strong antimicrobial activity [8,9,10]. ZnO NPs exhibit greater hepatotoxicity than conventional ZnO, with enhanced Zn^2+^ release under acidic lysosomal conditions and more severe oxidative stress, mitochondrial damage, and calcium overload [7]. The excessive environmental accumulation of ZnO NPs can lead to ecological harm and present significant public health risks [11,12]. Previous studies reported that ZnO NPs can also induce hepatotoxicity, nephrotoxicity, neurotoxicity, carcinogenicity, teratogenicity, and mutagenicity in humans and animals via the food chain or direct uptake [13,14,15,16]. For example, Grasso et al. reported that ZnO NPs have the potential to bioaccumulate in marine organisms, and seafood could be an important uptake route of ZnO NPs for human, then posing a toxic risk [12].

In liver tissues, it has been reported that the exposure to ZnO NPs in mammals can induce oxidative stress, DNA damage, apoptosis, and inflammatory response [17]. Almansour et al. reported that ZnO NP exposure at 2 mg/kg for 21 days can induce marked hepatoxic effects, which was evidenced by marked histopathological changes, including sinusoidal dilation, hepatocyte necrosis, chromatin fragmentation, Kupffer cell proliferation, and inflammatory cell infiltration within hepatic lobules and portal veins in rats [18]. Very recently, several studies showed that ZnO NP exposure can induce ferroptosis, pyroptosis and autophagy in the hepatocytes or liver tissues of mammals via the activation of multiple signaling pathways, including mitogen-activated protein kinase (MAPK), Toll-like receptors (TLRs), nuclear factor kappa-light-chain-enhancer of activated B cells (NF-κB), and NOD-like receptor family pyrin domain containing 3 (NLRP3)/cysteine-aspartic acid protease 1 (caspase-1)/gasdermin D (GSDMD) [5,19]. A recent study also indicated transcription factor EB (TFEB) is a key endogenous protective target that negatively regulates pyroptosis through autophagy-lysosomal biogenesis [20].

In the present study, we identified PF, a naturally occurring monoterpene glycoside, which can effectively attenuate ZnO NP exposure-induced cytotoxicity in HepG2 cells in vitro. The substantial preclinical evidence indicates that PF possesses anti-inflammatory and antioxidant properties [21]. Additionally, it was reported that the total glycoside of paeony containing PF (its account is >40%) had been widely used for the treatment of rheumatoid arthritis, systemic lupus erythematosus, psoriasis, and allergic contact dermatitis in more than 1000 hospitals in China with high safety [22,23]. PF has also been shown to regulate TFEB through multiple signaling pathways, including nuclear factor YA (NF-YA)-dependent transcriptional upregulation [24], AMP-activated protein kinase (AMPK)/Unc-51 like autophagy activating kinase (ULK1)-mediated autophagy activation [25], and Sirtuin 1 (SIRT1)-PTEN induced putative kinase 1 (PINK1)/Parkin-mediated mitophagy [26]. In this study, based on above-mentioned findings, we hypothesized that PF may attenuate ZnO NPs-induced hepatotoxicity by modulating the TFEB-mediated autophagy-lysosomal function. Our results, for the first time, reveal that PF can directly interact with SIRT1, then deacetylate the TFEB, and promote autophagy-lysosome biogenesis, finally effectively attenuating ZnO NPs-induced pyroptosis. Our research provides new insights into the molecular mechanisms of ZnO NPs hepatotoxicity. It also highlights that PF is an effective protective agent against ZnO NPs-induced hepatoxicity.

## 2. Materials and Methods

### 2.1. Chemicals and Reagents

ZnO NPs (>97%, 50 nm) were purchased from Sigma-Aldrich (St. Louis, MO, USA) and our previous studies have characterized their physicochemical properties [5]. PF (≥98%, P101691) was purchased from Aladdin Company (Shanghai, China). ZnO NPs were prepared as a 10 mg/mL stock solution by dispersing 10 mg of ZnO NPs in 1 mL DMSO, followed by sonication in an ultrasonic cleaner for 30 min, and the stock was stored at 4 °C and sealed with parafilm. Before each experiment, the stock solution was diluted with cell culture medium to the working concentration and sonicated again for 30 min. PF was dissolved in high-glucose DMEM to prepare a 100 mg/mL stock solution, which was then filter-sterilized and diluted with DMEM containing 10% FBS to working concentrations (100, 200, and 400 μg/mL) immediately before use. Unless otherwise stated, all other chemicals and reagents used in this study were of analytical grade.

### 2.2. Cell Culture

The HepG2 cells were purchased from Shanghai EKBioscience Biotechnology Co., Ltd. (Shanghai, China). HepG2 cell lines overexpressing TFEB stably (OV-TFEB) and TFEB knockout cell lines (KO-TFEB) were achieved in our last research [20]. Cells were cultured in DMEM with high glucose (Gibco, Grand Island, NY, USA), supplemented with 10% (*v*/*v*) FBS, 100 U/mL penicillin, and 100 µg/mL streptomycin (Gibco, Grand Island, NY, USA). Cells were maintained in a humidified incubator at 37 °C with 5% CO_2_.

### 2.3. Cell Viability and Lactate Dehydrogenase (LDH) Emission

The cell counting kit-8 (CCK)-8 assay was performed to evaluate cell metabolic activity. Briefly, HepG2 cells were seeded into 96-well plates at a density of 1 × 10^5^ cells/well. This density ensured the cells were in the logarithmic growth phase at the time of treatment, providing optimal metabolic activity and reproducibility for the CCK-8 assay, also falling within the linear detection range recommended by the manufacturer), and the cells were treated with ZnO NPs at 20 μg/mL or PF at 100, 200, or 400 μg/mL for 24 h, 48 h and 72 h. The negative control for all experiments consisted of cells treated with the vehicle solution (0.2% DMSO in culture medium) without ZnO NPs or PF, as the ZnO NP stock solution contained 0.2% DMSO as a dispersant, and this final solvent concentration was verified to have no cytotoxic effects on HepG2 cells. Subsequently, 100 μL of CCK-8 working solution (diluted in DMEM) was added to each well, followed by incubation at 37 °C for 1 h. Absorbance was measured at 450 nm using a microplate reader to determine cell viability. The working concentration of ZnO NPs was selected based on preliminary dose–response experiments, which identified the concentration causing approximately 50% cell viability reduction (IC_50_). This concentration was used for all subsequent mechanistic assays to ensure consistent and comparable experimental conditions across different detection platforms. The LDH kit was used to assist in the assessment of pyroptosis. After the positive control hole was established and the cells were treated according to the instructions, the culture supernatant was collected and transferred to a new 96-well plate to determine the LDH release ratio.

### 2.4. Immunofluorescence Staining

HepG2 cells (500 μL) were seeded into 24-well plates at a density of 5 × 10^4^ cells per well and treated as indicated. This density was chosen specifically to meet the technical requirements of immunofluorescence microscopy: it ensured that cells were well-spaced and individually distinguishable, allowing for clear visualization of subcellular localization and fluorescence intensity changes at the single-cell level. After treatment, cells were fixed with 4% paraformaldehyde for 30 min at room temperature, followed by three washes with PBS. Permeabilization was carried out using 0.5% Triton X-100 for 15 min. Cells were then blocked with 5% BSA in PBS for 2 h at room temperature. Subsequently, cells were incubated overnight at 4 °C with the primary antibody diluted in 5% BSA. After washing, cells were incubated with fluorescence-conjugated secondary antibody (1:500) for 2 h at room temperature in the dark. Nuclei were counterstained with DAPI, and coverslips were mounted using an anti-fade mounting medium. Images were acquired using a fluorescence microscope (Leica, Wetzlar, Germany).

### 2.5. Analysis of Cellular Fluorescence Probes

After 24 h of normal culture, the cells (2 mL) in a 6-well plate (2 × 10^5^ cells/well) were divided into four groups: control, PF (at 400 μg/mL), ZnO NPs (at 20 μg/mL), PF (at 400 μg/mL) + ZnO NPs (at 20 μg/mL) (co-incubation). The cell density was chosen to ensure optimal confluence for accurate fluorescence quantification, particularly given the varying specificity and quenching susceptibility of different fluorescent probes. TUNEL/DAPI staining: after treatment, cells were fixed with 4% paraformaldehyde (1 mL) for 30 min, permeabilized with 0.5% Triton X-100, and then subjected to TUNEL staining (100 µL reagent/well, 60 min, dark) to detect DNA fragmentation. The nuclei were counterstained with DAPI.

For LysoTracker staining, cells were treated with a 1 mL LysoTracker probe for 30 min, which has been diluted 10,000 times by the DMEM culture solution. DCFH-DA Staining: To measure intracellular reactive oxygen species (ROS), cells were loaded with DCFH-DA (1:1000 dilution in PBS) and incubated at 37 °C for 20 min in the dark. Fluorescence imaging was performed using a fluorescence microscope (Leica, Germany) or flow cytometry (BD Biosciences, San Jose, CA, USA).

### 2.6. Scanning Electron Microscopy (SEM) Observation

Cellular morphological alterations indicative of pyroptosis were examined by SEM. In brief, cells were treated with ZnO or co-treated with or without PF for 24 h. Then, cells were fixed overnight at 4 °C with pre-cooled 2.5% glutaraldehyde. After washing with PBS, cells were post-fixed with 1% osmium tetroxide for 1 h at room temperature. Samples were then dehydrated through a graded ethanol series (30%, 40%, 50%, 60%, 70%, 80%, 90%, and 100%), with two 10 min incubations at each concentration. Ethanol was subsequently replaced with isoamyl acetate, and critical-point drying was performed. Dried specimens were mounted on conductive adhesive, sputter-coated with gold, and imaged using a Hitachi S-4100 SEM system (Hitachi, Tokyo, Japan).

### 2.7. Molecular Docking

The 3D structure of SIRT1 (PDB ID: 4ZZJ) was obtained from the RCSB Protein Data Bank. The protein structure was prepared using AutoDock Tools 1.5.7 by removing co-crystallized ligands, water molecules, and extraneous ions. Polar hydrogens were added, and the resulting receptor was saved in PDBQT format. The ligand structure of PF was retrieved from the TCMSP database in MOL2 format. Its geometry was optimized and energy-minimized with Chem3D 2019 to obtain the lowest-energy conformation. The ligand was then processed in AutoDock Tools to assign Gasteiger charges, define rotatable bonds, and set torsional degrees of freedom, and finally converted to PDBQT format. Molecular docking was performed using AutoDock Vina 1.2.3. A grid box of 60 × 60 × 60 Å covering the entire protein was used to enable exhaustive sampling of potential binding sites. Docking poses with calculated binding affinities ≤ −5.0 kcal/mol were retained as indicative of favorable binding. The most stable docked complex was visualized and analyzed for intermolecular interactions using PyMOL 2.5.2 at a 2.0 Å interaction distance threshold.

### 2.8. Molecular Dynamics (MD) Simulations

MD simulations were performed using the GROMACS 2019.6 under periodic boundary conditions. The Amber14SB all-atom force field was used for the protein and ligand, and the TIP3P water model for the solvent. Both the apo-SIRT1 and PF-bound SIRT1 complex systems were energy-minimized using the Steepest Descent algorithm for 5000 steps to remove steric clashes. Following minimization, a 100 ps canonical (NVT) equilibration was conducted, with the temperature maintained at 300 K using the V-rescale thermostat. Subsequently, production runs of 100 ns were performed under an isothermal-isobaric (NPT) ensemble, with pressure maintained at 1 bar using the Parrinello-Rahman barostat. Covalent bonds involving hydrogen atoms were constrained with the LINCS algorithm, enabling an integration time step of 2 fs. Long-range electrostatic interactions were treated using the particle mesh Ewald (PME) method, and short-range non-bonded interactions were truncated at 10 Å. Trajectories were analyzed using GROMACS tools to evaluate root-mean-square deviation (RMSD), radius of gyration (Rg), and intermolecular hydrogen bonds. Structural visualization and interaction profiling were performed with VMD to characterize conformational dynamics and ligand-binding stability.

### 2.9. Surface Plasmon Resonance (SPR)

The sensor chip was activated with a 1:1 mix of 50 mM N-hydroxysuccinimide (NHS) and 200 mM 1-ethyl-3-(3-dimethylaminopropyl) carbodiimide (EDC). The SIRT1 protein was prepared at a concentration of 50 μg/mL in a 10 mM sodium acetate buffer and immobilized on the chip at 10 μL/min for a duration of 7 min. To block the remaining reactive groups, a 1 M solution of ethanolamine at pH 8.5 was employed. The final coupling density of SIRT1 reached 13,053.0 resonance units (RU). PF was serially diluted to eight analytical concentrations (0, 1.5625, 3.125, 6.25, 12.5, 25, 50, and 100 μM). Interaction assays ran at 30 μL/min with a 90 s association and 120 s dissociation phase. Binding kinetics were monitored in real time and subsequently analyzed with the BIAcore T200 evaluation software (version 3.1).

### 2.10. SIRT1 Enzymatic Coupling Reaction

The SIRT1-mediated deacetylation reaction was quantified through a coupled enzymatic assay. In brief, SIRT1 hydrolyzes NAD^+^ to generate nicotinamide, which is subsequently converted to ammonia by nicotinamidase PncA. Ammonia then reacts with α-ketoglutarate and NAD(P)H in the presence of glutamate dehydrogenase (GDH), yielding NAD(P)^+^. This sequence of reactions leads to a reduction in NAD(P)H absorbance at 340 nm. Prior to initiating the reaction, all reagents, with the exception of NAD^+^, were combined and pre-incubated at 37 °C for a duration of 10 min. The reaction commenced upon the addition of NAD^+^, and absorbance at 340 nm was monitored every 30 s for 30 cycles using a microplate reader. The linear phase of absorbance change (steepest slope) was selected for analysis. Reaction rates were calculated via the Lambert–Beer law: A = ε⋅c⋅d, where A = absorbance change, ε = molar extinction coefficient (6.22 mM^−1^cm^−1^), c = concentration change in NAD(P)H, and d = optical path length (0.3 cm). Deacetylation rates (μM/s) were determined by subtracting the baseline reaction rate (NAD+-free control) from experimental group values.

### 2.11. Cellular Thermal Shift Assay (CETSA)

A CETSA was performed to assess target engagement between PF and SIRT1, based on the principle that ligand binding increases protein thermal stability. HepG2 cells were seeded in 6-well plates at 2 × 10^5^ cells/well in 2 mL per well and were treated with PF or DMEM (control), washed with PBS, and harvested by trypsinization. Single-cell suspensions were centrifuged at 1500 rpm for 5 min, and pellets were resuspended in 200 μL DMEM. Aliquots were heated in a PCR thermal cycler at a gradient of 45–65 °C or maintained at room temperature. After heating, samples were centrifuged and the supernatants removed. Cell pellets were lysed with RIPA buffer on ice for 10 min. Resulting lysates were analyzed by Western blotting as described in Section 2.12.

### 2.12. Western Blotting

Western blotting was performed according to our previous description [27]. In brief, HepG2 cells were seeded in 6-well plates at 2 × 10^5^ cells/well in 2 mL per well and treated with ZnO NPs (20 μg/mL) and/or PF (100, 200, 400 μg/mL) for 24 h before protein extraction. And the cells were lysed with commercial RIPA buffer containing 1% PMSF (Beyotime, Nantong, China). Lysates were sonicated and centrifuged at 14,000× *g* for 15 min at 4 °C to obtain supernatants. The protein concentration was determined using a BCA protein assay kit (Beyotime, Nantong, China). Samples were denatured with loading buffer at 100 °C for 10 min, and equal amounts (30 µg) were separated by SDS-PAGE (80 V stacking gel, 120 V separating gel). Proteins were transferred to PVDF membranes, which were then blocked with 5% skim milk and incubated sequentially with primary antibodies including rabbit or mouse against TFEB, NLRP3, LC3, ASC, GSDMD, SIRT1, IL-1β, CTSB, CTSD, LAMP2, IL-18, caspase-1 (all 1:1000 dilution; Cell Signaling Technology, Danvers, MA, USA), and β-actin (1:1000 dilution, Protein Tech, Dallas, TX, USA) for overnight at 4 °C. Peroxidase-conjugated goat anti-rabbit or anti-mouse IgG (1:5000) (Protein Tech, Dallas, TX, USA) were used as the secondary antibodies. After TBST washing, bands were detected using ECL (Beyotime, Nantong, China) and quantified with Image J, and the densitometry results were normalized relative to the β-actin bands.

### 2.13. qRT-PCR Analysis

Cells were seeded in 6-well plates at 2 × 10^5^ cells/well in 2 mL per well and treated with ZnO NPs (20 μg/mL) and/or PF (400 μg/mL) for 24 h before RNA extraction. Total RNA was extracted from treated hepatocytes using the TRIzol reagent according to the manufacturer’s protocol (Invitrogen, Carlsbad, CA, USA). Subsequently, 1 µg of total RNA was reverse-transcribed into cDNA using a PrimeScript™ RT Reagent Kit (Takara, Dalian, China). RT-qPCR was performed on a LightCycler^®^ 480 System (Roche, Basel, Switzerland) using SYBR^®^ Green Premix Pro Taq HS qPCR Kit (Accurate Biology, Hunan, China). The cycling conditions were 95 °C for 30 s, followed by 40 cycles of 95 °C for 5 s and 60 °C for 30 s. Relative expression was calculated using the 2^−ΔΔCt^ method and normalized to the internal control β-actin. The primer sequences used in this study are listed in Supplementary Table S2.

### 2.14. Co-Immunoprecipitation

HepG2 cells were seeded in 6-well plates at 2 × 10^5^ cells/well and cultured for 24 h to reach approximately 70% confluence. Transfection was performed using the Lipofectamine 6000 reagent (Beyotime, China) according to the manufacturer’s instructions. Briefly, for each well, 2 μg of plasmid DNA (SIRT1 overexpression or Flag-TFEB) was diluted in 100 μL of Opti-MEM, and 4 μL of Lipofectamine 6000 was diluted in 100 μL of Opti-MEM. The two solutions were mixed, incubated at room temperature for 15 min, and then added dropwise to the cells. After 6 h, the medium was replaced with fresh complete DMEM, and cells were cultured for an additional 24 h. Then cells were subjected to treatment with 10 μM EX527 or 400 μg/mL PF. Subsequently, the cells were lysed using a lysis buffer supplemented with 1 mM PMSF and deacetylase inhibitors. The lysates were incubated with anti-Flag magnetic beads at 4 °C. After three washes, bound proteins were eluted in 2 × SDS buffer. Immunoprecipitated complexes were evaluated by Western blot using anti-acetylated lysine, anti-Flag, and anti-SIRT1 antibodies.

### 2.15. Data Analysis

Data analysis and graph creation were conducted using GraphPad Prism 10. Statistical analysis was conducted using the One-way ANOVA method, with results presented as mean ± standard deviation (S.D.). All experiments were performed as at least three independent biological replicates, each with three technical replicates. A *p*-value less than 0.05 was considered as significant difference.

## 3. Results

### 3.1. PF Is an Effective Protective Agent Against ZnO NP-Induced Cytotoxicity and Oxidative Damage in HepG2 Cells

**Figure 1:**
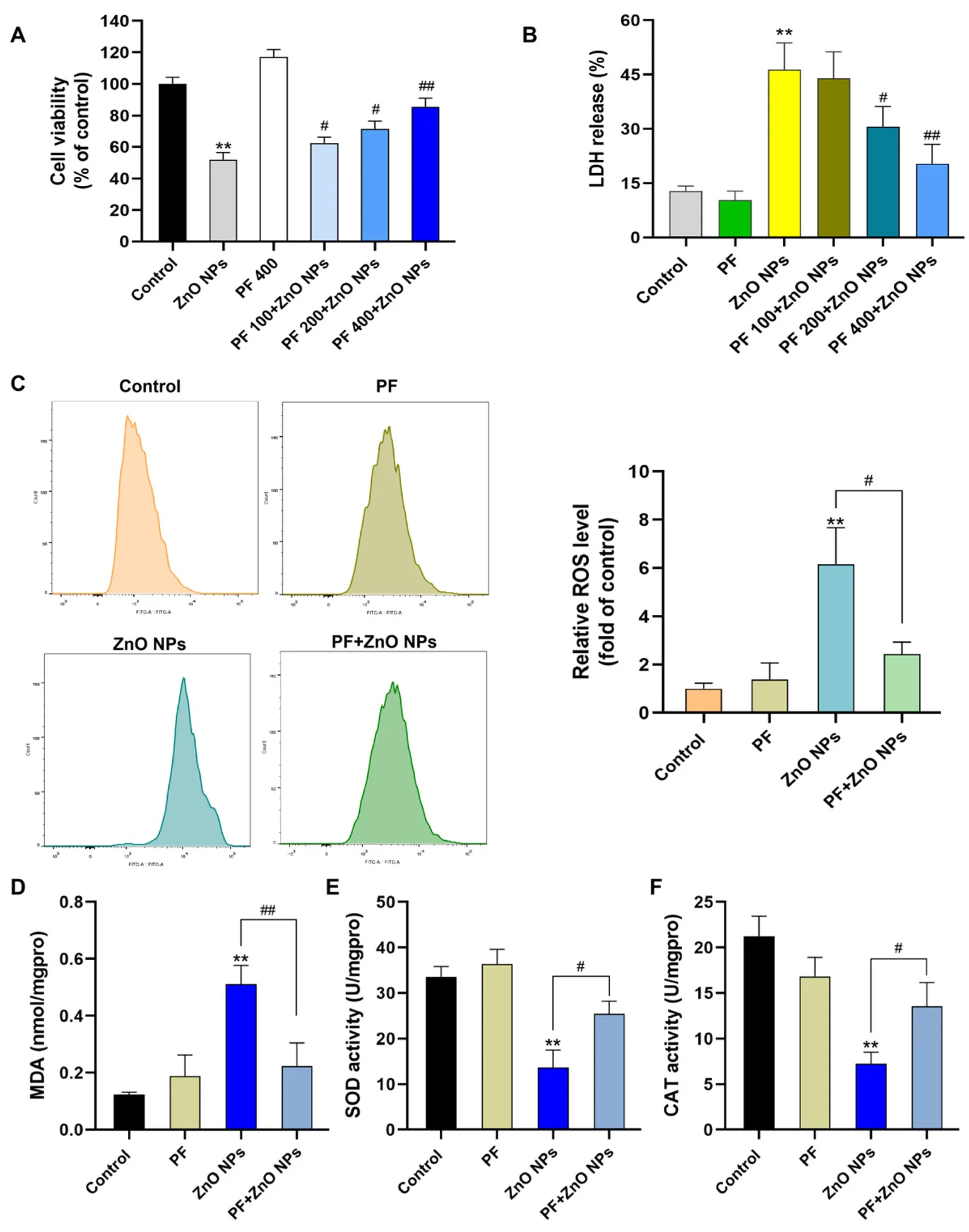
PF is an effective protective agent against ZnO NP-induced cytotoxicity and oxidative damage in HepG2 cells. (**A**) PF dose-dependently attenuates ZnO NP-induced cytotoxicity in HepG2 cells. (**B**) The levels of LDH. (**C**) The intracellular ROS levels. (**D**–**F**) Effects of PF on ZnO NP-induced MDA, SOD and CAT. Data are presented as mean ± SD from three independent experiments (*n* = 3). The negative control was DMEM supplemented with 10% FBS containing 0.2% DMSO. ** *p* < 0.01, compared with control; # *p* < 0.05, ## *p* < 0.01, compared with ZnO NPs alone.

The hydrodynamic diameter, polydispersity index (PDI), and zeta potential of ZnO NPs were comparable in the absence and presence of PF (Supplementary Table S1), confirming that PF co-incubation did not alter the aggregation state or colloidal stability of the nanoparticles. In HepG2 cells, ZnO NP treatment at 20 μg/mL for 24 h significantly decreased the cell viabilities to 50%. PF treatment at 100, 200 and 400 μg/mL dose-dependently revised ZnO NP exposure-induced loss in cell viability (Figure 1A). Furthermore, PF supplementation also significantly decreased the LDH release in a dose-dependent manner, compared to that in the control group (Figure 1B). Furthermore, we assessed the protective effects of PF supplementation on ZnO NP-induced oxidative stress. As shown in Figure 1C, the flow cytometry analysis utilizing DCFH-DA demonstrated that PF effectively inhibited ROS production when compared to the treatment group receiving only ZnO NPs (*p* < 0.01; Figure 1C). The malondialdehyde (MDA) concentration in total protein decreased from 0.51 nmol/mg in the ZnO NP-only treatment group to 0.22 nmol/mg (*p* < 0.01; Figure 1D). Additionally, the concentrations of superoxide dismutase (SOD) and catalase (CAT) increased from 13.66 U/mg protein and 7.27 U/mg protein to 25.41 U/mg protein and 13.53 U/mg protein (both *p* < 0.01), respectively (Figure 1E,F). These findings indicate that PF has the potential to mitigate ZnO NP-induced cytotoxicity and oxidative stress.

To assess whether PF provides sustained protection, cell viability was extended to 48 and 72 h. At 48 h, ZnO NPs alone caused nearly complete cell death, whereas PF co-treatment slightly preserved viability; at 72 h, both ZnO NPs alone and PF + ZnO NP groups showed almost no viable cells (Supplementary Figure S1A,B). In addition, PF did not alter FluoZin-3 fluorescence (Supplementary Figure S1C) or the kinetics of intracellular total zinc accumulation over 24 h (Supplementary Figure S1D), indicating that PF neither chelates zinc nor reduces intracellular zinc burden. HepG2 cells were exposed to ZnO NPs (20 μg/mL) for 24 h, after which the medium was removed and replaced with fresh medium containing PF (400 μg/mL), EDTA Ca-salt (50 μM), or PF + EDTA Ca-salt, respectively, for an additional 24 h. EDTA Ca-salt alone showed no significant protective effect (Supplementary Figure S4), whereas PF alone or PF + EDTA Ca-salt treatments both significantly improved cell viability after ZnO NP removal, indicating that PF acts through intracellular repair mechanisms rather than extracellular zinc chelation.

### 3.2. PF Supplementation Alleviates ZnO NP-Induced Cell Pyroptosis

**Figure 2:**
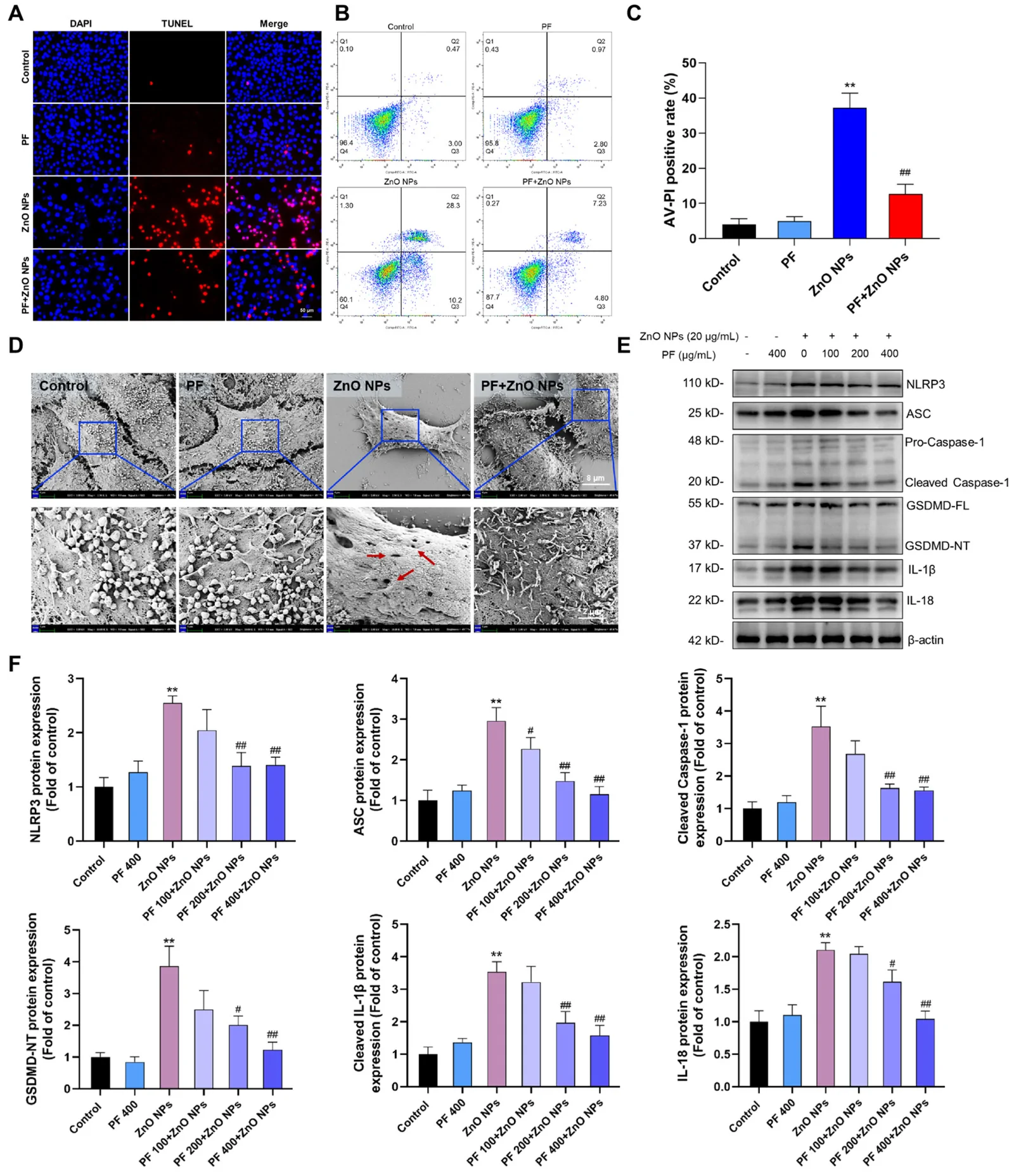
PF supplementation alleviates ZnO NP-induced cell pyroptosis. (**A**) Fluorescence microscopy images of TUNEL staining in cells treated with ZnO NPs in the presence or absence of PF. (**B**,**C**) Flow cytometry quantification of Annexin V/PI staining showing suppression of plasma membrane rupture by 400 μg/mL PF. (**D**) Representative SEM images observing cell morphological alterations. Red arrows indicate cell swelling and membrane perforation. Scale bars = 8 or 2 μm. (**E**,**F**) Western blot analysis of inflammasome assembly and pyroptosis-related proteins, including NLRP3, ASC, caspase-1, GSDMD, IL-1β, and IL-18. Data are presented as mean ± SD from three independent experiments (*n* = 3). The negative control was DMEM supplemented with 10% FBS containing 0.2% DMSO. ** *p* < 0.01, compared with control; # *p* < 0.05, ## *p* < 0.01, compared with ZnO NPs alone.

As shown in Figure 2A, PF supplementation at 400 μg/mL significantly reduced the TUNEL-positive rate. Consistent results were also showed in Figure 2B. This indicated that PF supplementation alleviated ZnO NP-induced plasma membrane rupture. We further observed the morphology changes using an SEM. As shown in Figure 2D, PF supplementation attenuated cell swelling and membrane perforation. Additionally, we examined the expression of several pyroptosis-related proteins and inflammasome assembly proteins. As shown in Figure 2E,F, ZnO NP exposure markedly upregulated the expression of pyroptosis-related proteins and inflammasome assembly proteins (including NLRP3, ASC, cleaved caspase-1, GSDMD-NT, IL-1β, and IL-18). Correctly, our data suggested that PF supplementation could effectively alleviate ZnO NP-induced pyroptosis.

### 3.3. PF Promotes Autophagy-Lysosome Biogenesis in ZnO NP-Exposed Cells

**Figure 3:**
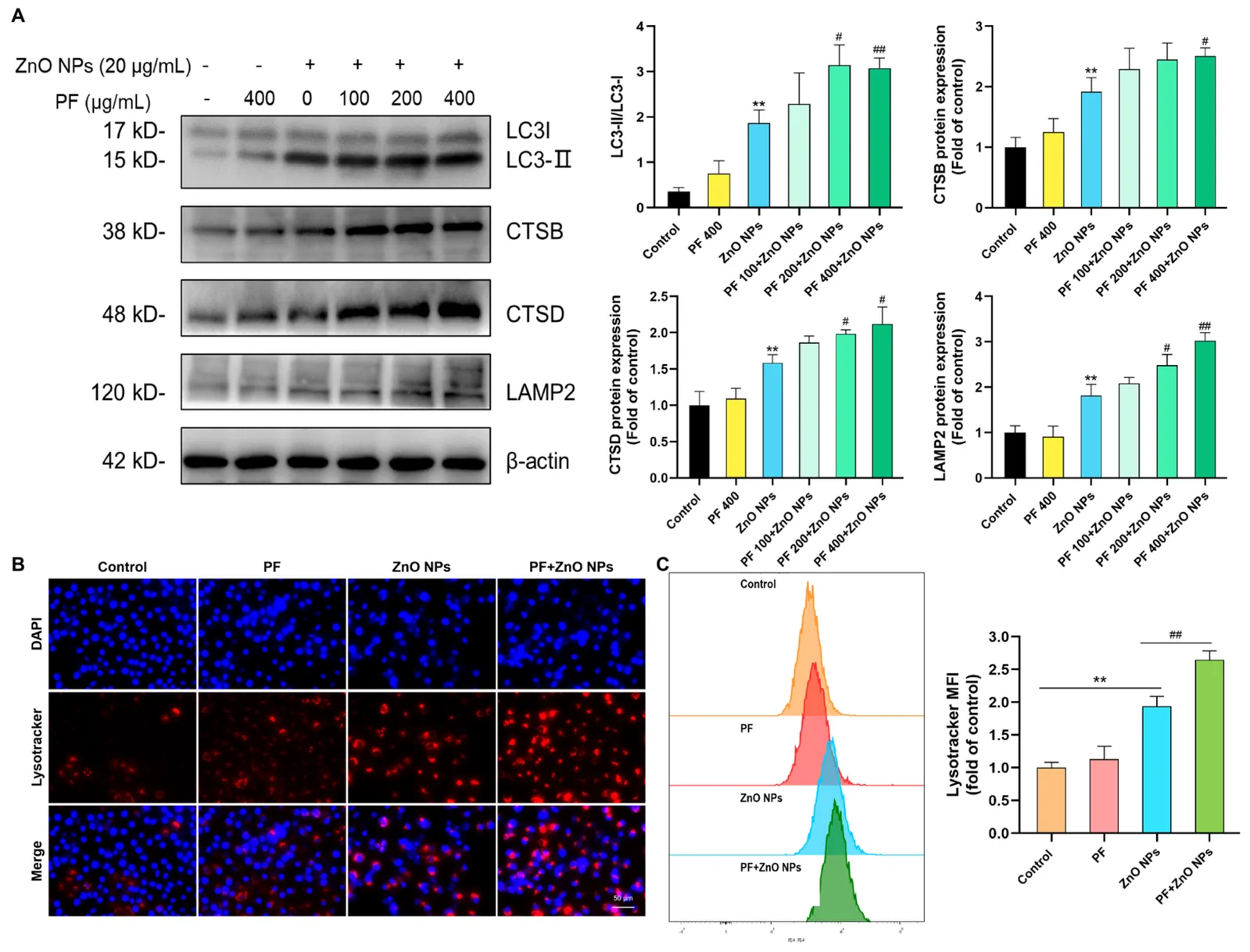
PF promotes autophagy-lysosome biogenesis in ZnO NP-exposed cells. (**A**) Western blot analysis of autophagy and lysosomal marker proteins, including LC3, CTSB, CTSD, and LAMP2. (**B**) Fluorescence microscopy images of LysoTracker Red staining, employed to assess lysosomal mass and functional activity. (**C**) Quantitative analysis of the mean fluorescence intensity (MFI) of LysoTracker using flow cytometry. Data are presented as mean ± SD from three independent experiments (*n* = 3). The negative control was DMEM supplemented with 10% FBS containing 0.2% DMSO. ** *p* < 0.01, compared with control; # *p* < 0.05, ## *p* < 0.01, compared with ZnO NPs alone.

We further found that PF treatment significantly increased the levels of mRNA expression enriched in the autophagy-lysosome biogenesis pathway (Supplementary Figure S2). We also analyzed the expression of related protein in the autophagy-lysosome biogenesis pathway. As illustrated in Figure 3A, compared to ZnO NP-alone treatment group, PF treatment markedly increased the LC3-II/LC3-I ratio. Furthermore, PF treatment significantly increased the expression of lysosomal marker proteins, including CTSB, CTSD, and LAMP2. Consistently, LysoTracker staining was used to observe the effects of PF on lysosomes. As shown in Figure 3B,C, PF increased both the proportion and activity of lysosomes, with the average fluorescence intensity of LysoTracker rising, compared to ZnO NP-alone treatment. These results indicated that PF treatment significantly activated the autophagy-lysosome biogenesis pathway.

### 3.4. TFEB Is Required for PF-Mediated Cytoprotection Against ZnO NPs Through Autophagy-Lysosome Activation and Pyroptosis Suppression

**Figure 4:**
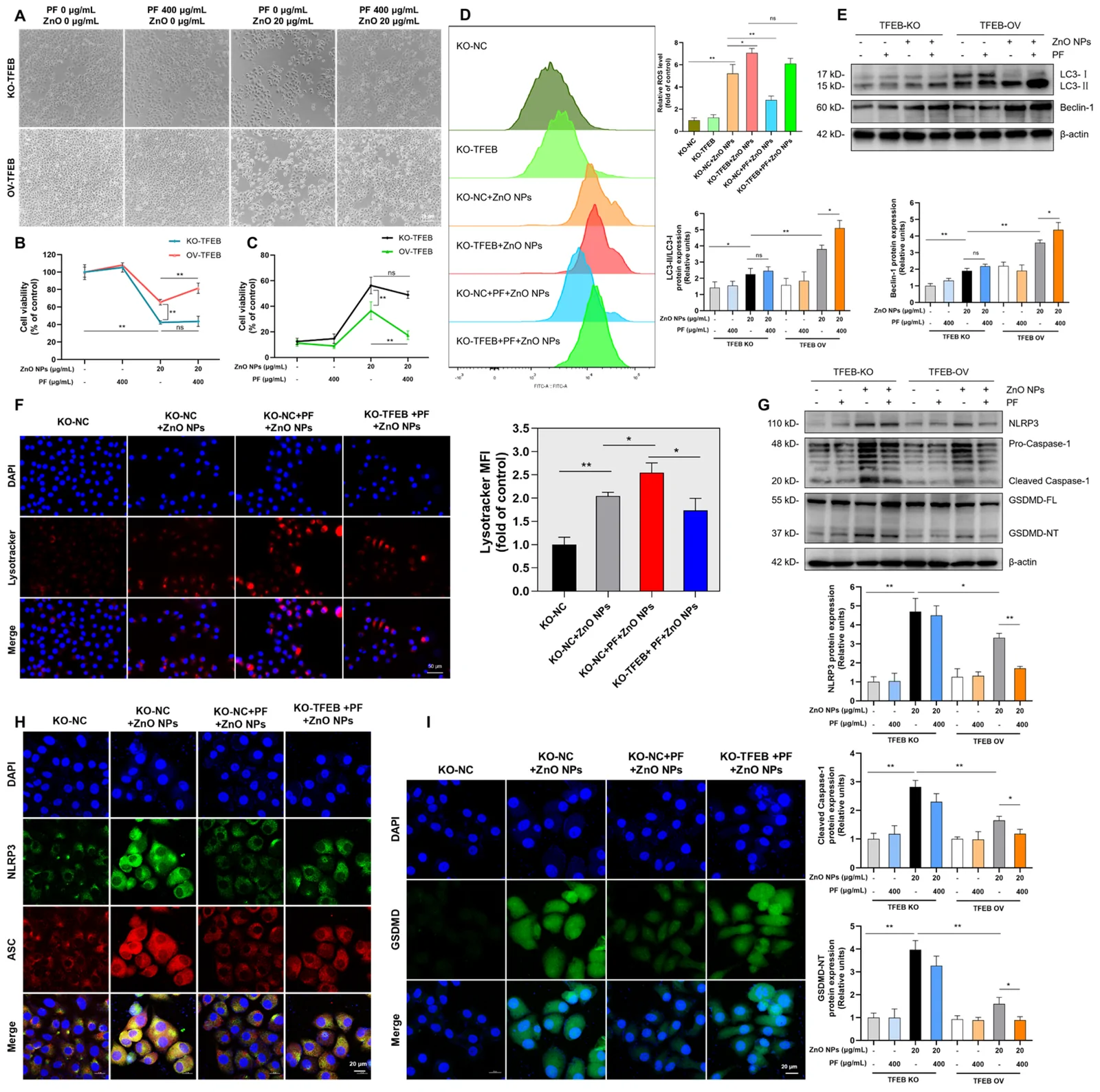
TFEB is required for PF-mediated cytoprotection against ZnO NPs through autophagy-lysosome activation and pyroptosis suppression. (**A**) The cell morphology was observed under light microscope in OV-TFEB and KO-TFEB cells. (**B**) Cell viability rescue by PF (400 μg/mL) in ZnO NPs (20 μg/mL)-exposed TFEB-modified HepG2 cells. (**C**) LDH release assay confirms TFEB-dependent membrane protection by PF. (**D**) Relative ROS level by flow cytometry. Intracellular ROS suppression by PF requires TFEB expression. (**E**) Autophagy flux and Beclin-1 expression are enhanced by PF only in OV-TFEB cells. (**F**) LysoTracker probe fluorescence. Scale bar: 50 μm (**G**) Pyroptosis-related protein expression. (**H**) Immunofluorescence of ASC and NLRP3. Scale bar: 20 μm (**I**) Immunofluorescence of GSDMD. Scale bar: 20 μm. Data are presented as mean ± SD from three independent experiments (*n* = 3). The negative control was DMEM supplemented with 10% FBS containing 0.2% DMSO. * *p* < 0.05, ** *p* < 0.01.

To validate TFEB as a downstream target mediating the cytoprotective effects of PF, the KO-TFEB and OV-TFEB cell lines were treated with PF under exposure to 20 μg/mL ZnO NPs. As demonstrated in Figure 3B and Figure 4A, under these conditions, the viability of the OV-TFEB cell line (65.6%) was significantly higher than that of the KO-TFEB cell line (42.5%). Treatment with 400 μg/mL PF significantly restored the viability of the OV-TFEB cell line to 81.6% but did not significantly restore the viability of the KO-TFEB cell line. Additionally, TFEB knockout weakened the inhibitory effect of PF on LDH release (Figure 4C). As shown in Figure 4D, when TFEB was not knocked out, PF significantly alleviated the ZnO NP-induced increase in ROS. However, TFEB knockout inhibited the effect of PF, and the decrease in ROS was not significant. These findings suggested that TFEB can influence PF’s intervention in cytotoxicity and oxidative stress.

As shown in Figure 4E, compared to KO-TFEB, the expression of LC3-II/LC3-I and Beclin-1 were elevated under TFEB overexpression conditions. Following PF intervention, the expression of LC3 and Beclin-1 was further significantly augmented in the OV-TFEB cell line, while no significant effect was observed on autophagy marker expression in the KO-TFEB cell line (Figure 4E). As revealed in Figure 4F, PF significantly increased the MFI of LysoTracker and elevated the lysosomal ratio when TFEB was not knocked out. However, TFEB knockout weakened the effect of PF on lysosome biogenesis, as evidenced by a significant decrease in the MFI of LysoTracker. These results suggest that TFEB was involved in regulating PF’s effects on the autophagy-lysosome pathway. Regardless of TFEB knockout or overexpression, ZnO NPs were found to increase the activation of NLRP3, cleaved caspase-1, and GSDMD-NT (Figure 4G). Compared to TFEB knockout, these markers were significantly downregulated in the OV-TFEB cell line. Furthermore, TFEB knockout weakened PF’s intervention in the pyroptosis pathway. In the OV-TFEB cell line, PF effectively inhibited the increase in NLRP3, cleaved caspase-1, and GSDMD-NT induced by ZnO NPs (Figure 4G). However, in the KO-TFEB cell line, PF did not significantly alleviate the upregulation of NLRP3 and GSDMD-NT (Figure 4G). Laser confocal microscopy was used to observe the colocalization of inflammasomes and GSDMD expression. The results showed that the overlapping fluorescence intensity of NLRP3 and ASC, as well as the fluorescence intensity of GSDMD, were reduced due to PF intervention (Figure 4H,I). However, TFEB knockout reversed this signaling change. These findings indicate that TFEB plays a role in mediating the effects of PF on ZnO NP-induced pyroptosis and serves as a crucial target for the protective effects of PF against hepatotoxicity.

### 3.5. PF Activates the SIRT1/mTOR Pathway, Facilitating the Nuclear Translocation of TFEB

**Figure 5:**
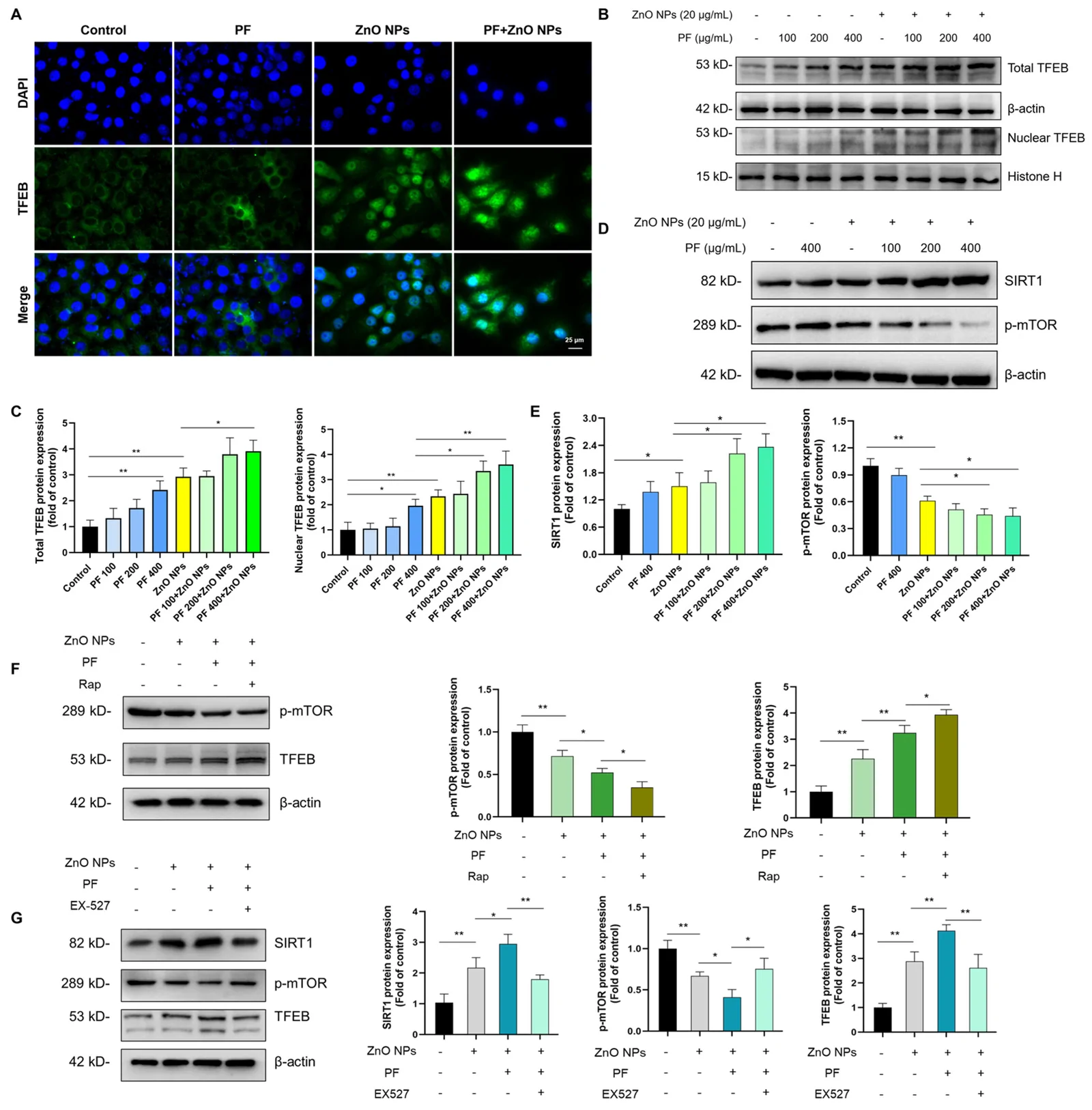
PF activates the SIRT1/mTOR pathway, facilitating the nuclear translocation of TFEB. (**A**) Immunofluorescence confirms PF-induced TFEB nuclear translocation. Scale bar: 25 μm. (**B**,**C**) PF enhances TFEB expression and nuclear translocation in ZnO NP-exposed HepG2 cells. (**D**,**E**) PF modulates SIRT1-mTOR-TFEB pathway. (**F**) Rapamycin (Rap, 10 μM) were co-treated into HepG2 cells for 24 h with PF or ZnO NPs. Protein expression of TFEB and p-mTOR were measured by Western blot. (**G**) 10 μM EX527 were co-treated into HepG2 cells for 24 h with PF or ZnO NPs. Protein expression of TFEB, SIRT1, and p-mTOR were measured by Western blot. Data are presented as mean ± SD from three independent experiments (*n* = 3). The negative control was DMEM supplemented with 10% FBS containing 0.2% DMSO. * *p* < 0.05, ** *p* < 0.01.

Compared to treatment with ZnO NPs alone, PF significantly increased both the total intracellular TFEB protein expression and the nuclear protein expression, indicating that PF promotes TFEB upregulation while also facilitating its nuclear translocation (Figure 5B,C). Immunofluorescence analysis of TFEB expression and cellular sublocalization revealed that PF further increased the green fluorescence intensity of TFEB aggregated in the nucleus, indicating that PF promoted TFEB activation (Figure 5A). Additionally, compared to treatment with ZnO NPs alone, treatment with PF at concentrations of 200 and 400 μg/mL significantly enhanced the protein expression of SIRT1, while the expression of phosphorylated mTOR was markedly inhibited (Figure 5D,E). These results suggested that the SIRT1/mTOR/TFEB pathway may be involved in the protective effects of PF against ZnO NP-induced hepatotoxicity. To verify the upstream and downstream relationships of SIRT1, mTOR and TFEB, this study used 10 μM Rapamycin (Rap) to inhibit mTOR activity in the PF-treated cell model. The blockade of p-mTOR under PF intervention further upregulated TFEB expression (Figure 5F). Inhibition of SIRT1 expression using 10 μM EX-527 revealed that blocking SIRT1 further upregulated p-mTOR and simultaneously downregulated TFEB under PF intervention (Figure 5G). These results suggested that PF regulated autophagy-pyroptosis through the SIRT1/mTOR/TFEB pathway to improve its intervention in ZnO NP-induced hepatotoxicity. We also examined the dynamics of MT expression, the principal zinc-buffering system. ZnO NP exposure time-dependently upregulated MT1A and MT2A mRNA, peaking at 12 h and declining by 24 h. PF co-treatment did not significantly alter this induction profile (Supplementary Figure S3), indicating that PF does not enhance MT-mediated zinc buffering.

### 3.6. Molecular Docking and MD Simulation System of PF and SIRT1

**Figure 6:**
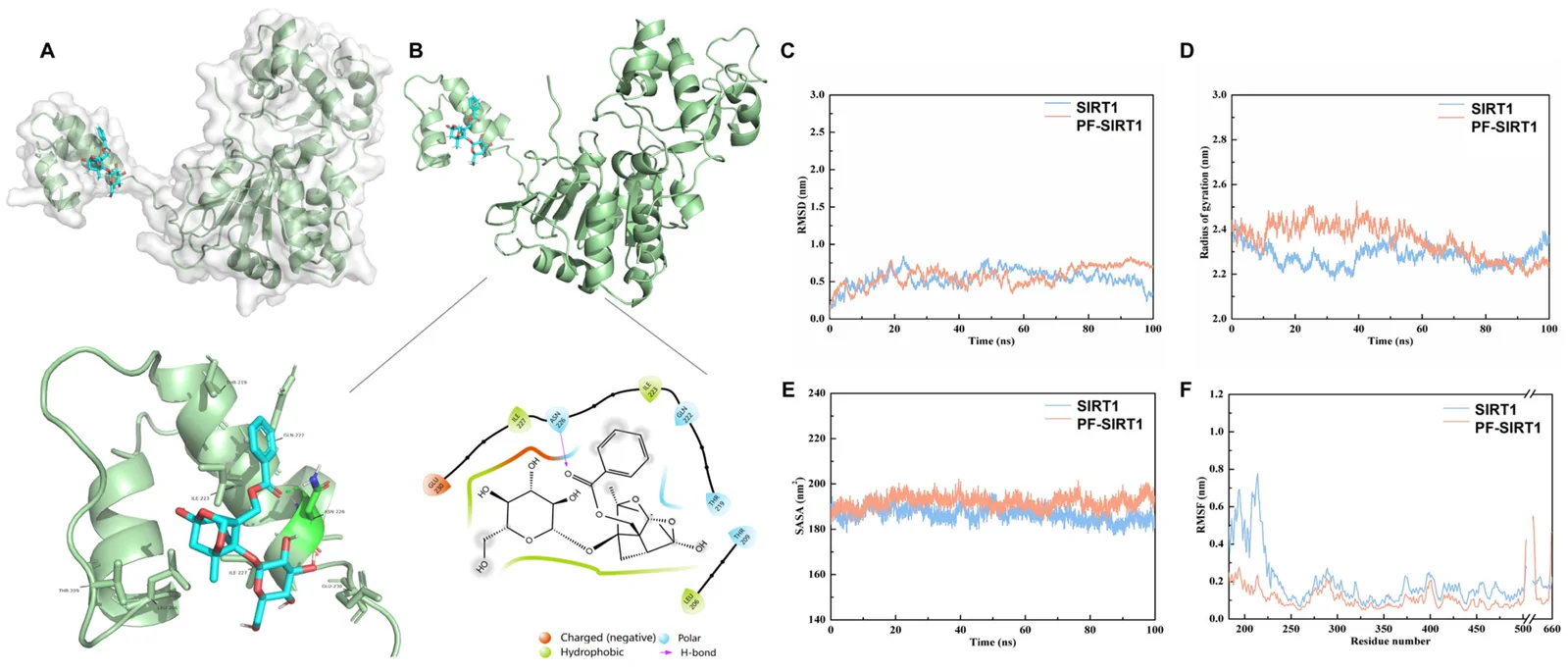
Molecular docking and MD simulation system of PF and SIRT1. (**A**) Predicted binding pose of PF within the STAC-binding domain of SIRT1 (PDB: 4ZZJ). (**B**) Interaction details of PF-SIRT1. Hydrogen bond between PF’s carbonyl oxygen and ASN226; hydrophobic contacts with LEU206, ILE223, ILE227. Change in RMSD (**C**), Rg (**D**) and SASA (**E**) with time and distribution of RMSF (**F**).

We assume that PF exerts a protective effect by activating SIRT1 and then sirt1 deacetylates TFEB. Therefore, we carried out the PF-SIRT1 molecular docking. In light of the previously reported SIRT1 agonist binding sites, molecular docking of PF-SIRT1 (PDB: 4ZZJ) was performed within the STAC-binding domain (SBD) located in the N-terminal region [28]. Using AutoDock Vina, the binding energy at the agonist site was calculated to be −5.228 kcal/mol, indicating a stable interaction. The interaction mode of PF-SIRT1 was depicted in Figure 6A. Analysis revealed that the oxygen atom near the phenylcyclocarbonyl group of PF forms hydrogen bonds with the ASN226 residue of the SIRT1 protein. Additionally, PF engages in hydrophobic interactions with the hydrophobic amino acids LEU206, ILE223, and ILE227 of SIRT1 (Figure 6B). MD simulation was employed to predict the molecular recognition process between PF and SIRT1, using RMSD and Rg as reference indicators of the system stability. RMSD reflected the degree of deviation of all protein backbone atoms from their reference positions. As shown in Figure 6C, the RMSD values fluctuated less significantly after 60 ns for both the SIRT1 and PF-SIRT1 systems, with average RMSD values of 0.545 ± 0.082 nm and 0.657 ± 0.104 nm, respectively. The increase in RMSD may be related to intermolecular interactions. Rg indicates the expansiveness and compactness of polymers. After 60 ns, the Rg values of both systems stabilized (fluctuations < 0.2 nm), with Rg values of 2.277 ± 0.037 nm and 2.274 ± 0.042 nm, respectively, showing no significant changes in polymer size (Figure 6D). Solvent-accessible surface area (SASA) was used to predict the interactions between the complex and surrounding solvent molecules. It was observed that SASA fluctuations remained minimal for both systems, with average SASA values of 191.868 ± 2.933 nm^2^ and 184.877 ± 2.640 nm^2^ for the PF-SIRT1 and SIRT1 systems after 60 ns, indicating stable binding (Figure 6E). RMSF reflects the flexibility of amino acid residues, with lower values indicating greater molecular stability. As shown in Figure 6F, the overall flexibility of the SIRT1 protein decreased upon binding with PF. These parameters suggested that the binding of PF and SIRT1 tends toward a relatively stable state.

### 3.7. MD Reveal PF-Induced Allosteric Modulation of SIRT1 Through Stable Binding at the Catalytic-Proximal Site

**Figure 7:**
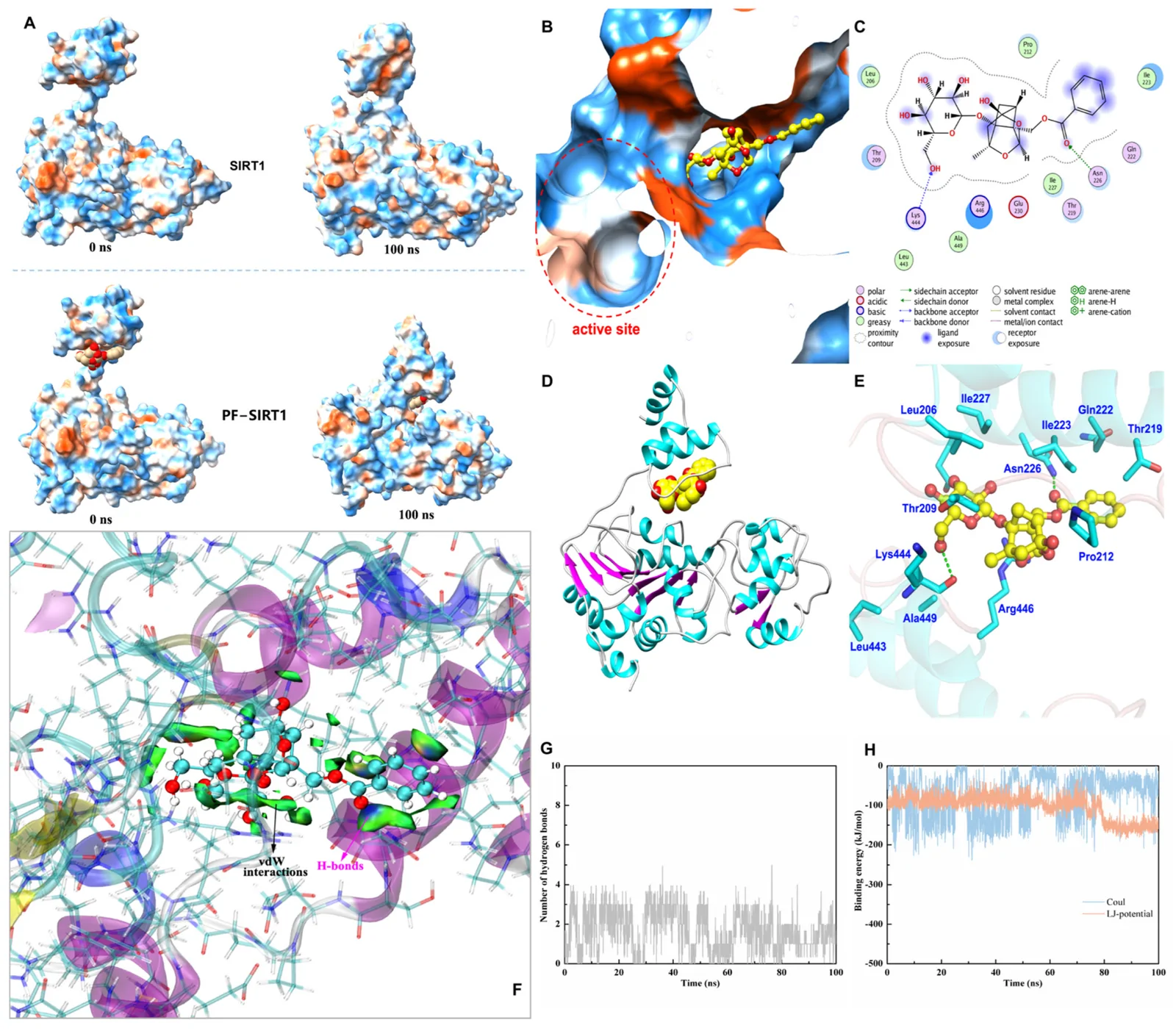
Molecular dynamics reveal PF-induced allosteric modulation of SIRT1 through stable binding at the catalytic-proximal site (**A**) Conformational dynamics of SIRT1. (**B**) Binding mode of hydrophilic (blue) and hydrophobic (orange) surfaces. (**C**) 2D binding model. (**D**,**E**) 3D binding model (dotted line: hydrogen bond). (**F**) Analysis of weak interaction of PF-SIRT1 by IGM. (**G**) Number of hydrogen bonds. (**H**) Binding energy of PF-SIRT1 system.

The direct targeting of PF to SIRT1 was further predicted by analyzing the structure of the simulated complex. First, the hydrophilic and hydrophobic surfaces during the simulation were examined. As illustrated in Figure 7A, the structural alterations of the protein within the PF-SIRT1 system were more significant compared to those observed in the SIRT1 system. During the MD simulation, the SBD region of SIRT1 gradually approached the deacetylase catalytic domain, a motion pattern that might be related to the activation of SIRT1 enzymatic function. Figure 7B showed that PF binds within a cavity of the SIRT1 protein. This cavity was located near the catalytic active site of SIRT1, potentially influencing its catalytic function directly. Additionally, PF formed hydrogen bonds with ASN226 and LYS444, and engages in hydrophobic interactions with hydrophobic amino acids, including LEU206, PRO212, ILE223, ILE227, ALA449 and LEU443 (Figure 7C–E). Figure 7F revealed relatively few hydrogen bond interactions and a widespread distribution of van der Waals interactions. An examination of the hydrogen bond count and binding energy data within the PF-SIRT1 system revealed that the number of hydrogen bonds remained relatively stable throughout the simulation (Figure 7G). The electrostatic interaction (Coul) between PF and SIRT1 was relatively weak, while the van der Waals interaction (LJ-potential) exhibits minimal fluctuation during the simulation and stabilizes after 80 ns. The average values of Coul and LJ-potential were −50.168 ± 23.708 kJ/mol and −154.092 ± 10.852 kJ/mol, respectively (Figure 7H). Based on all simulation results, it could be predicted that the binding of PF and SIRT1 achieves a stable state, primarily relying on van der Waals forces, hydrophobic interactions, and hydrogen bonding.

### 3.8. Validation of PF-SIRT1 Binding and Functional Activation Driving TFEB Deacetylation

**Figure 8:**
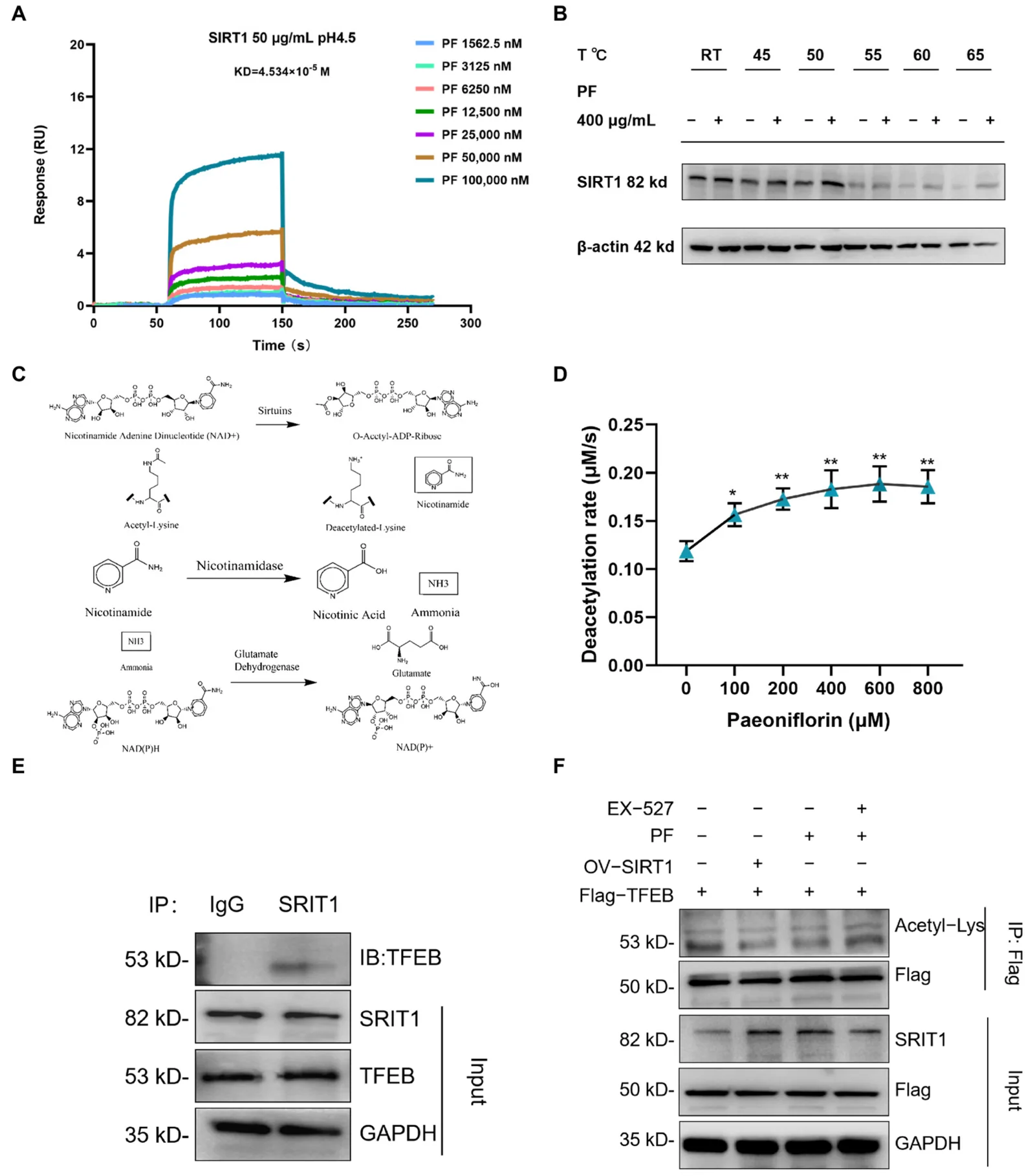
Validation of PF-SIRT1 binding and functional activation driving TFEB deacetylation. (**A**) Analysis of PF-SIRT1 binding affinity via SPR. (**B**) CETSA analysis for thermal stability of SIRT1 protein. PF-bound SIRT1 exhibits enhanced thermal stability. (**C**) Principle of SIRT1 enzyme coupling reaction. (**D**) Effect of PF on SIRT1 deacetylase rate. (**E**) Endogenous TFEB co-immunoprecipitated with SIRT1. (**F**) TFEB acetylation was detected by IP-WB. Data are presented as mean ± SD from three independent experiments (*n* = 3). The negative control was DMEM supplemented with 10% FBS containing 0.2% DMSO. * *p* < 0.05, ** *p* < 0.01, compared with control.

SPR was further used to confirm the binding affinity between PF and SIRT1. The results showed that SIRT1 protein could be stably immobilized at a concentration of 50 μg/mL, and PF displayed a typical binding–dissociation curve with SIRT1. The calculated affinity constant (KD) was 45.34 μM (Figure 8A), indicating that PF could directly bind to SIRT1. The CETSA results showed that while the proteins in all groups gradually degraded under stepwise temperature increases, the intracellular SIRT1 protein in the PF-treated group exhibited higher thermal stability compared to the untreated control group, suggesting that PF may bind to SIRT1 to enhance the structural stability of this receptor protein (Figure 8B). The SIRT1-mediated deacetylation reaction was quantified through a coupled enzymatic assay (Figure 8C). The deacetylase activity results for SIRT1 demonstrated that PF could increase the deacetylation rate of SIRT1 on its substrate TFEB peptide in a concentration-dependent manner (Figure 8D). Endogenous Co-IP using a SIRT1-specific antibody confirmed TFEB presence in immunoprecipitates, demonstrating a physical interaction between TFEB and SIRT1 (Figure 8E). To determine whether SIRT1 modulated TFEB acetylation, we treated HepG2 cells with either SIRT1 overexpression plasmids, PF or EX-527. Immunoprecipitation analysis revealed that SIRT1 overexpression significantly reduced TFEB acetylation levels, whereas EX-527 treatment markedly increased TFEB acetylation. These findings indicated that PF directly binded SIRT1, stabilized its conformation, enhanced SIRT1 deacetylase activity toward TFEB substrate, and ultimately reduced TFEB acetylation through validated SIRT1-TFEB physical interaction.

## 4. Discussion

The present study identifies PF as a pharmacological modulator of the SIRT1-TFEB axis in ZnO NP-induced hepatocyte injury. In details, PF reduced oxidative damage and canonical NLRP3 inflammasome-mediated pyroptosis, restored autophagy-lysosome biogenesis, and promoted TFEB nuclear accumulation. Genetic manipulation further demonstrated that TFEB was required for the cytoprotective effects of PF. At the molecular level, PF directly interacted with SIRT1, increased its deacetylase activity toward TFEB, and reduced TFEB acetylation. Together with the observed inhibition of mTOR signaling, these events facilitated TFEB activation and strengthened lysosomal and autophagic capacity. These findings from Figure 1, Figure 2, Figure 3, Figure 4, Figure 5, Figure 6, Figure 7 and Figure 8 extend previous observations that TFEB coordinates autophagy and pyroptosis during ZnO NP-induced hepatotoxicity and establishes a target-centered mechanism through which PF regulates this response.

Oxidative stress and inflammatory cell death are closely interconnected components of ZnO NP-induced hepatotoxicity. Following cellular uptake, ZnO NPs can disrupt redox homeostasis and organelle function, generating intracellular danger signals that promote NLRP3 inflammasome assembly and caspase-1 activation. Activated caspase-1 subsequently cleaves gasdermin D, producing the pore-forming GSDMD-N-terminal fragment and facilitating the maturation and release of IL-1β and IL-18. The previous work from this group identified oxidative stress-related canonical pyroptosis as an important mechanism of ZnO NP-induced liver injury [20]. In the present study, ZnO NP exposure induced cell swelling, membrane disruption, LDH release, NLRP3 and ASC accumulation, caspase-1 activation, GSDMD cleavage, and increased IL-1β and IL-18 expression. The convergence of morphological, biochemical, and molecular evidence therefore supports the involvement of canonical pyroptosis rather than nonspecific loss of membrane integrity alone. In this present study, PF markedly attenuated these responses while reducing ROS and MDA accumulation and restoring SOD and CAT activities. These results are consistent with the established antioxidant and anti-inflammatory properties of PF in experimental liver injury [29,30,31]. Nevertheless, the present findings suggest that its protective activity is not limited to direct redox regulation. PF suppressed the NLRP3-caspase-1-GSDMD cascade in parallel with restoration of the autophagy-lysosome system, indicating that improved intracellular quality control may contribute to the removal of damaged organelles and inflammasome-activating signals. The antioxidant and anti-pyroptotic effects of PF may therefore represent functionally connected consequences of enhanced autophagic and lysosomal homeostasis.

Autophagy-lysosome dysfunction is increasingly recognized as a critical determinant of nanoparticle toxicity. Depending on particle properties and exposure conditions, nanoparticles may accumulate within lysosomes, alter lysosomal acidity and membrane integrity, impair cargo degradation, and disrupt autophagic flux [32]. Such disturbances can facilitate the persistence of damaged mitochondria and oxidized macromolecules, thereby sustaining ROS generation and inflammasome activation [32]. TFEB functions as a master transcriptional regulator of lysosomal biogenesis and autophagy and is therefore positioned at the interface between organelle quality control and inflammatory cell death [33]. Previous studies demonstrated that impaired TFEB activity aggravates liver injury by compromising lysosomal biogenesis and autophagic clearance, whereas restoration of TFEB activity improves cellular adaptation to metabolic and toxicological stress [6,25]. In the present study, PF increased the LC3-II/LC3-I ratio and the expression of lysosomal proteins, including CTSB, CTSD, and LAMP2, while enhancing LysoTracker-associated lysosomal signals (shown in Figure 3). More importantly, the effects of PF were strongly dependent on TFEB status. TFEB overexpression improved cell survival, autophagy-related protein expression, and lysosomal responses, and enhanced the suppression of pyroptosis by PF. Conversely, TFEB knockout substantially weakened the ability of PF to restore cell viability, reduce ROS and LDH release, promote autophagy-lysosome activity, and inhibit NLRP3 and GSDMD activation. These genetic data move the proposed role of TFEB beyond a correlative change in protein expression and support TFEB as an essential mediator of PF-induced cytoprotection.

It has been demonstrated that PF can promote autophagy in several pathological contexts [34,35,36]. For instance, PF can activate LKB1/AMPK-dependent autophagy during intestinal ischemia–reperfusion injury, enhances autophagy-associated signaling in LPS-challenged microglial cells, and promotes KLF4-dependent mitophagy in macrophages [25,37,38]. PF or its derivatives have also been reported to inhibit caspase-1 and GSDMD-associated pyroptosis in astrocytes, microglia, and macrophages [29,39]. The present study integrates these previously separate observations by demonstrating that PF-mediated activation of the TFEB-dependent autophagy-lysosome system is mechanistically associated with suppression of pyroptosis in ZnO NP-exposed hepatocytes.

A major advance of this study is the identification of SIRT1 as a direct molecular target involved in PF-mediated TFEB activation (Figure 5, Figure 6, Figure 7 and Figure 8). Previous network pharmacology and docking analyses suggested several candidate targets of PF, including AKT1, MAPKs, mTOR, and SIRT1, but these predictions did not establish direct target engagement or functional activation. Here, molecular docking and molecular dynamics simulations predicted a stable interaction between PF and a region of SIRT1 proximal to the reported STAC-binding domain. This computational prediction was subsequently supported by orthogonal experimental approaches. Surface plasmon resonance demonstrated direct PF-SIRT1 binding with a KD of 45.34 μM, while the cellular thermal shift assay showed that PF increased the thermal stability of intracellular SIRT1. Moreover, PF concentration-dependently enhanced SIRT1-mediated deacetylation of a TFEB substrate, and co-immunoprecipitation confirmed the physical association between SIRT1 and TFEB. The reduction in TFEB acetylation induced by PF or SIRT1 overexpression, together with its reversal by EX-527, provides a functional link between PF-SIRT1 engagement and TFEB modification. These findings are biologically relevant because acetylation is an important regulatory mechanism governing TFEB activity. SIRT1-mediated deacetylation of TFEB has been shown to promote lysosomal biogenesis and the clearance of intracellular protein aggregates [40]. Other natural compounds can also activate this regulatory axis. For example, berberine promotes SIRT1-dependent TFEB deacetylation and autophagy in macrophages, whereas resveratrol can enhance SIRT1 activity by stabilizing interactions between SIRT1 and selected substrates [41,42]. The present study extends this paradigm to PF and provides evidence spanning predicted structural interaction, biophysical binding, intracellular target engagement, enzymatic activation, substrate deacetylation, and downstream biological effects. However, because the precise PF-binding site was inferred computationally rather than resolved experimentally, PF should currently be described as a SIRT1-binding activator rather than definitively classified as a site-specific allosteric agonist.

TFEB activity is regulated not only by acetylation but also by phosphorylation. Under nutrient-rich conditions, lysosome-associated mTORC1 phosphorylates TFEB and promotes its cytoplasmic retention. Inhibition of mTORC1 permits TFEB dephosphorylation and nuclear translocation, resulting in transcriptional activation of lysosomal and autophagy-related genes [43,44,45]. In the present study, PF increased SIRT1 expression, reduced mTOR phosphorylation, and promoted TFEB nuclear accumulation. Pharmacological inhibition of SIRT1 with EX-527 restored mTOR phosphorylation and reduced TFEB expression under PF treatment, whereas rapamycin further enhanced TFEB activation. These findings support a model in which PF regulates TFEB through two complementary mechanisms: direct enhancement of SIRT1-dependent TFEB deacetylation and suppression of mTOR-dependent inhibitory signaling.

The relationship among SIRT1, mTOR, and TFEB is nevertheless likely to be more complex than a simple linear pathway. SIRT1 may indirectly restrain mTORC1 through metabolic and upstream signaling regulators, while TFEB-mediated lysosomal adaptation may in turn alter nutrient sensing and mTOR localization. The present results establish functional ordering among these proteins but do not fully determine whether PF-mediated mTOR inhibition is exclusively dependent on SIRT1 or whether PF also affects mTOR through parallel pathways. Accordingly, the data are most appropriately interpreted as supporting coordinated regulation of a SIRT1/mTOR/TFEB network, with SIRT1-dependent TFEB deacetylation representing the most directly validated molecular event.

The present findings also complement previous in vivo evidence showing that PF alleviates ZnO NP-induced liver injury by reducing pyroptosis and regulating the gut–liver axis [20]. The earlier study established systemic hepatoprotection, whereas the current work identifies a hepatocyte-intrinsic mechanism centered on direct SIRT1 engagement and TFEB-dependent autophagy-lysosome activation [20]. Taken together, our study combined with the previous study co-suggest that the effects of PF may operate at multiple biological levels: modulation of intestinal and systemic responses in vivo and restoration of intracellular quality-control pathways within hepatocytes. This multilevel activity may be particularly relevant to nanoparticle-induced injury, which involves direct cellular stress as well as inflammatory and metabolic communication among tissues. While our data indicate that PF does not act through zinc chelation or metallothionein induction, future studies may investigate whether PF influences the expression or activity of ZIPs and ZnTs. Nevertheless, the protective effects of PF are most likely mediated through SIRT1-dependent pathways, as demonstrated in other experimental models, including alleviation of pulmonary arterial hypertension via the TRIM24/SIRT1/NLRP3 axis and amelioration of reperfusion injury through SIRT1-PINK1/parkin-mediated mitochondrial autophagy [26,46]. These findings support a model in which PF enhances SIRT1/TFEB-mediated autophagy-lysosomal clearance rather than directly modulating zinc homeostasis.

## 5. Conclusions

**Figure 9:**
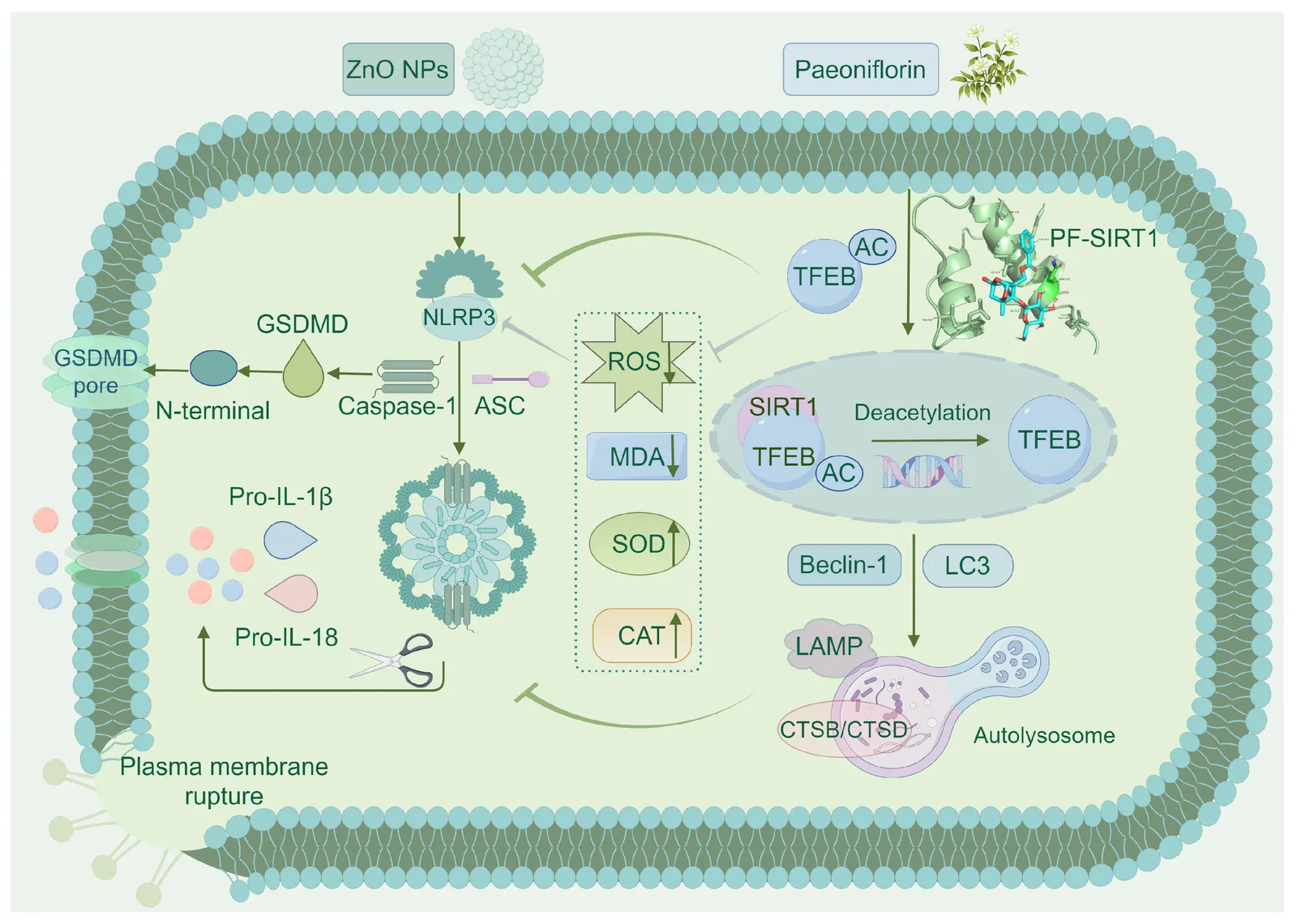
Schematic diagram of the SIRT1-TFEB axis underlying PF-mediated hepatoprotection against ZnO NP-induced toxicity (By Figdraw version 2.0).

In conclusion, this study establishes a mechanistic framework in which PF directly engages and activates SIRT1, promotes TFEB deacetylation and nuclear translocation, restores autophagy-lysosome biogenesis, and consequently limits NLRP3 inflammasome activation and pyroptosis in ZnO NP-exposed hepatocytes (Figure 9). The results connect molecular target engagement with post-translational regulation, organelle quality control, and inflammatory cell death. Although further validation in physiologically relevant models is required, the SIRT1-TFEB axis represents a promising target for developing protective strategies against nanomaterial-induced liver injury.

## Data Availability

All data are included in the manuscript. Data are available upon reasonable request.

## Supplementary Materials

The following supporting information can be downloaded at: https://www.mdpi.com/article/10.3390/antiox15101284/s1, Table S1: Hydrodynamic diameter, PDI, and zeta potential of ZnO NPs in complete cell culture medium in the absence or presence of PF; Table S2: Primer sequences used for qRT-PCR; Figure S1: Cell viability at 48 h and 72 h, FluoZin-3 fluorescence, and intracellular total zinc dynamics in HepG2 cells treated with ZnO NPs, PF, or PF + ZnO NPs; Figure S2: Effects of PF on mRNA expression of autophagy-lysosome-related genes in ZnO NPs-exposed HepG2 cells; Figure S3: Time-course of MT1A and MT2A mRNA expression in HepG2 cells treated with ZnO NPs, PF, or PF + ZnO NPs; Figure S4: PF promotes cell recovery after ZnO NP removal.
