# Supplementary material for: Paeoniflorin Attenuates Zinc Oxide Nanoparticle-Induced Pyroptosis in HepG2 Cells via the Activation of SIRT1/TFEB Signaling Pathway
Source: Antioxidants (Basel). 2026 Oct 3;15(10):1284. doi: 10.3390/antiox15101284 (PMC13700757; doi:10.3390/antiox15101284)
Supplement: Supplementary file 1 [file antioxidants-15-01284-s001.zip › antioxidants-4504345-supplementary.pdf]

Supplementary Methods

DLS measurements in complete cell culture medium

The hydrodynamic diameter, polydispersity index (PDI), and zeta potential of ZnO NPs (20 μg/mL) alone and PF (400 μg/mL) + ZnO NPs were measured in complete DMEM supplemented with 10% FBS using a dynamic light scattering instrument (DynaPro NanoStar, Wyatt, USA) and a zeta potential analyzer (Nano-ZS, Malvern, UK). Measurements were performed at 25°C. Data are presented as mean ± SD from three independent measurements.

FluoZin-3 AM assay

HepG2 cells were seeded into 6-well plates and treated with vehicle (0.2% DMSO), PF (400 μg/mL), ZnO NPs (20 μg/mL), or PF (400 μg/mL) + ZnO NPs for 24 h. After treatment, cells were washed once with HBSS and incubated with FluoZin-3 AM (1 μM) in HBSS at 37 °C for 30 min in the dark. Cells were then washed three times with HBSS to remove extracellular probe. Fluorescence images were acquired using a fluorescence microscope (Leica, Germany) with excitation/emission at 494/516 nm.

Intracellular total zinc measurement

HepG2 cells were seeded into 6-well plates and treated with vehicle (0.2% DMSO), PF (400 μg/mL), ZnO NPs (20 μg/mL), or PF (400 μg/mL) + ZnO NPs for 0, 3, 6, 12, and 24 h. After treatment, cells were washed three times with ice-cold, metal-free PBS to remove extracellular zinc, then lysed in 0.1% Triton X-100. Cell lysates were collected by scraping, and total zinc content was determined by flame atomic absorption spectrometry (FAAS). Zinc concentrations were normalized to total protein concentration measured by the BCA assay and expressed as μg Zn per mg protein.

Supplementary Table S1 Hydrodynamic diameter, PDI, and zeta potential in DMEM

Group	Hydrodynamic diameter	PDI	Zeta
ZnO NPs	171.86 ± 11.34 nm	0.28 ± 0.06	12.63 ± 0.52
PF+ZnO NPs	167.28 ± 12.69 nm	0.31±0.05	10.85 ± 0.48

Supplementary Table S2 Primer sequences

Gene Name	Gene Accession Number	Forward Primer (5′→3′)	Reverse Primer (5′→3′)
SIRT1	NM_001142498.2	TGTGTCATAGGTTAGGTGGTGA	AGCCAATTCTTTTTGTGTTCGTG
TFEB	NM_001271944.2	AAGGTTCGGGAGTATCTGTCTG	GGGTTGGAGCTGATATGTAGCA
LAMP1	NM_005561.4	CAGATGTGTTAGTGGCACCCA	TTGGAAAGGTACGCCTGGATG
LAMP2	NM_002294.3	GCACAGTGAGCACAAATGAGT	CAGTGGTGTGTATGGTGGGT
CTSB	NM_001908.5	ACAACGTGGACATGAGCTACT	TCGGTAAACATAACTCTCTGGGG
CTSD	NM_001909.5	TGCTCAAGAACTACATGGACGC	CGAAGACGACTGTGAAGCACT
CTSF	NM_003793.4	AGAGAGGCCCAATCTCCGT	GCATGGTCAATGAGCCAAGG
MCOLN1	NM_020533.3	TCTTCCAGCACGGAGACAAC	GCCACATGAACCCCACAAAC
ATP6V1H	NM_015941.4	CCGAGGATTCGCGAGATTTG	TGCTTCTCTTCAGGGCTTCG
ATP6V1A	NM_001690.4	TTATGAACGAGCAGGCAGGG	CACCAGGTGGAGAAACTGCT
LC3B	NM_022818.5	GAGAAGCAGCTTCCTGTTCTGG	GTGTCCGTTCACCAACAGGAAG
p62	NM_003900.5	GCACCCCAATGTGATCTGC	CGCTACACAAGTCGTAGTCTGG
Beclin 1	NM_003766.5	ACCTCAGCCGAAGACTGAAG	AACAGCGTTTGTAGTTCTGACA
ATG5	NM_004849.4	AAAGATGTGCTTCGAGATGTGT	CACTTTGTCAGTTACCAACGTCA
MT1A	NM_005946.3	CTCGAAATGGACCCCAACT	ATATCTTCGAGCAGGCGTGTC
MTA2	NM_005953.5	CCCGCTCCCAGATGTAAAGA	CAGCAGCTGCACTTGTCCG
β-actin	NM_001101.5	CGGGAAATCGTGCGTGAC	CAGGAAGGAAGGCTGGAAGAG

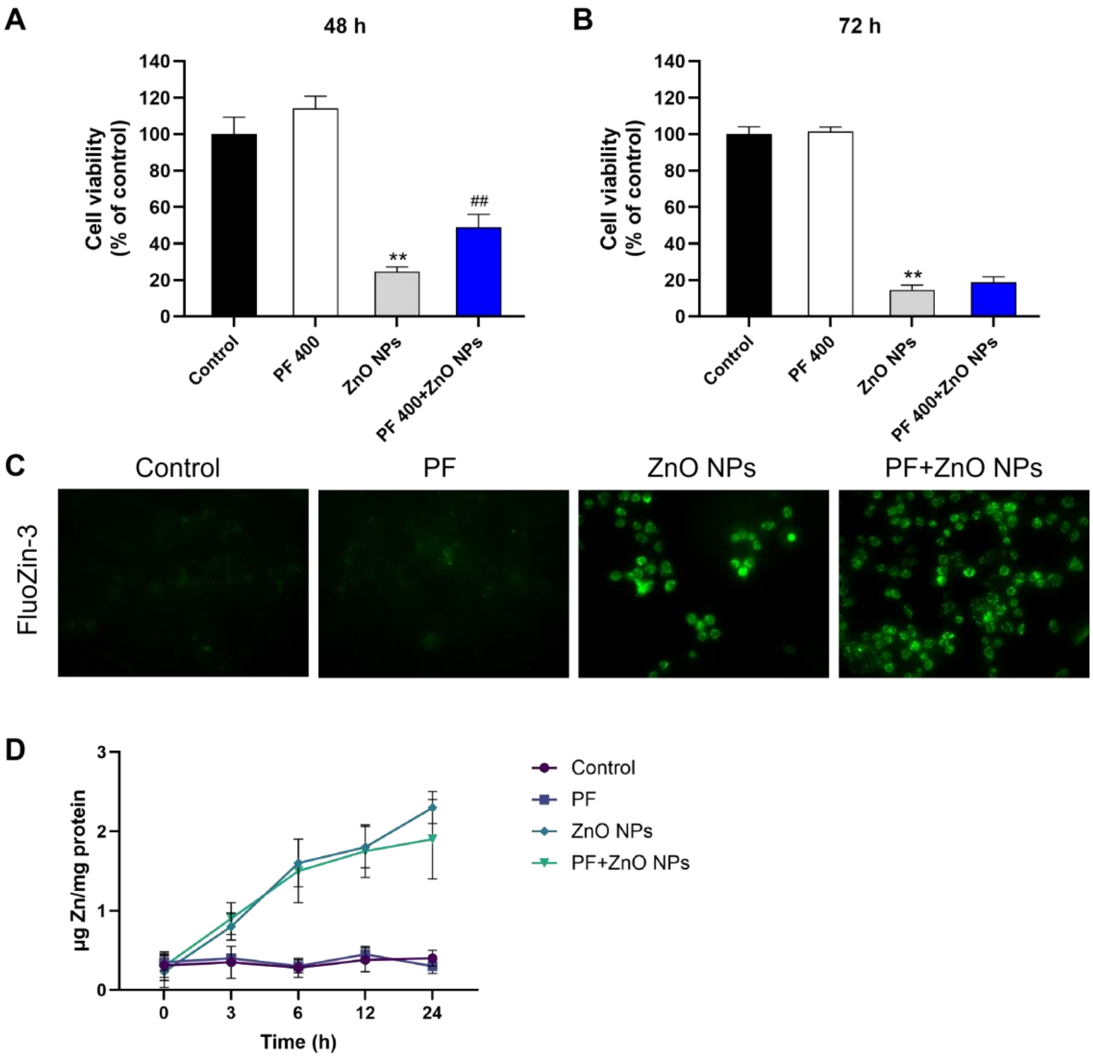

Supplementary Figure S1. (A, B) Cell viability of HepG2 cells treated with ZnO NPs (20 μg/mL), PF (400 μg/mL), or PF + ZnO NPs for 48 h (A) and 72 h (B). (C) FluoZin-3 fluorescence in ZnO NP-treated cells with or without PF. (D) Intracellular total zinc content measured by FAAS at 0, 3, 6, 12, and 24 h. Data are presented as mean ± SD from three independent experiments (n = 3). The negative control was DMEM supplemented with 10% FBS containing 0.2% DMSO. ** $p < 0.01$, compared with control; ## $p < 0.01$, compared with ZnO NPs alone.

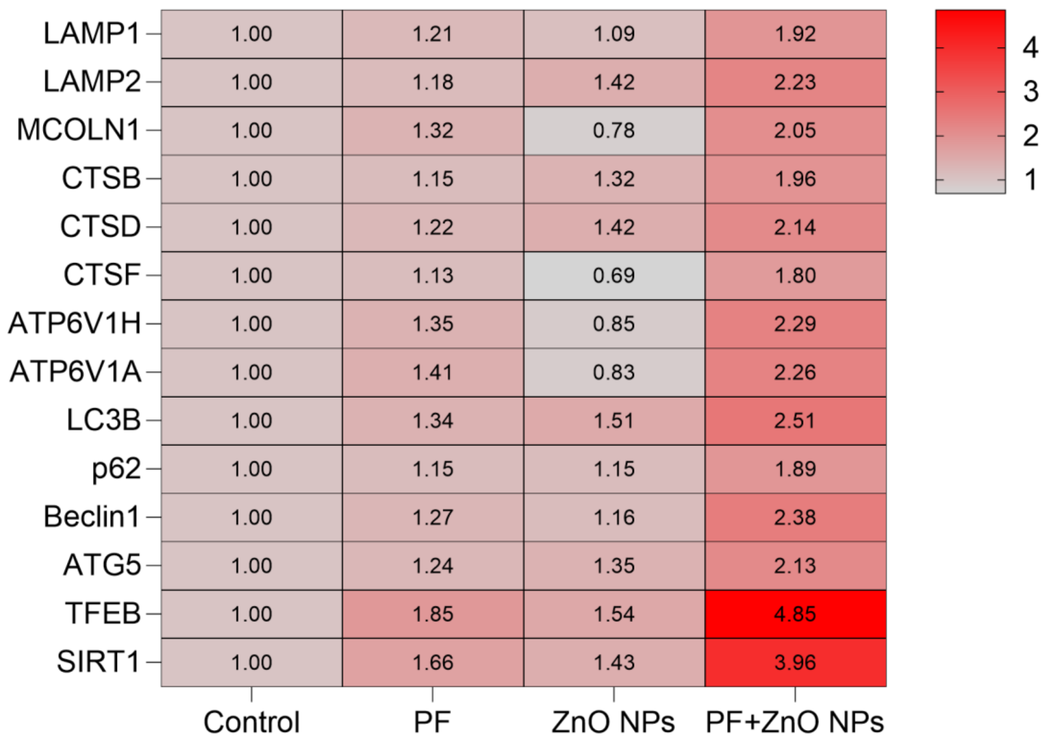

Supplementary Figure S2. Effects of PF on mRNA expression of autophagy-lysosome-related genes in ZnO NPs-exposed HepG2 cells.

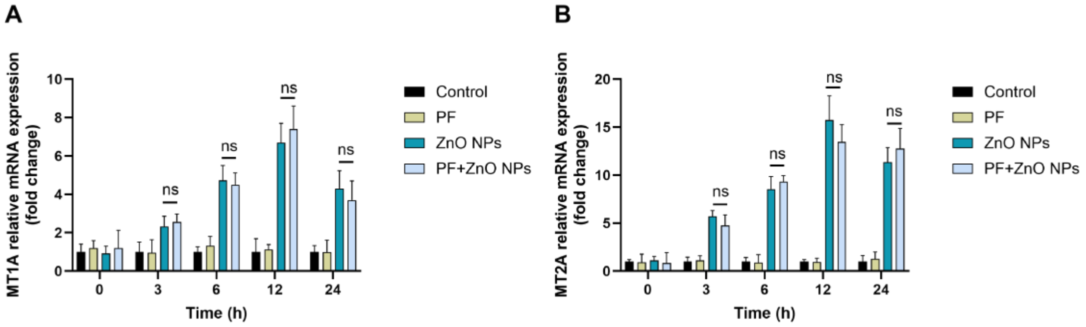

Supplementary Figure S3. Time-course of MTs mRNA expression in HepG2 cells treated with vehicle, ZnO NPs (20 μg/mL), PF (400 μg/mL), or PF + ZnO NPs for 0, 3, 6, 12, and 24 h. Relative mRNA expression of MT1A (A) and MT2A (B) was determined by qRT-PCR and normalized to β-actin using the $2^{-\Delta\Delta Ct}$ method. Data are presented as mean ± SD from three independent experiments.

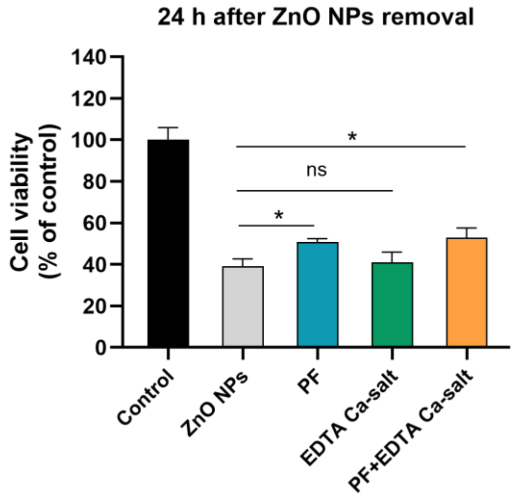

Supplementary Figure S4. PF promotes cell recovery after ZnO NPs removal. HepG2 cells were exposed to ZnO NPs (20 μg/mL) for 24 h, after which the ZnO NPs-containing medium was removed and replaced with fresh medium containing PF (400 μg/mL), EDTA Ca-salt (50 μM), or PF + EDTA Ca-salt. For additional 24 h, cell viability was assessed by CCK-8. Data are presented as mean ± SD from three independent experiments. * $p < 0.05$; ns, not significant.
